# Role of the p38 MAPK/C/EBPβ Pathway in the Regulation of Phenotype and IL-10 and IL-12 Production by Tolerogenic Bone Marrow-Derived Dendritic Cells

**DOI:** 10.3390/cells7120256

**Published:** 2018-12-07

**Authors:** Chantal Guindi, Alexandre Cloutier, Simon Gaudreau, Echarki Zerif, Patrick P. McDonald, Olga Tatsiy, Claude Asselin, Gilles Dupuis, Denis Gris, Abdelaziz Amrani

**Affiliations:** Immunology Division, Faculty of Medicine and Health Sciences and Centre de Recherche du CHUS, Université de Sherbrooke, 3001, 12th Avenue North, Sherbrooke, QC J1H 5N4, Canada; chantal.guindi@usherbrooke.ca (C.G.); alexander.cloutier@usherbrooke.ca (A.C.); sgaudreau@ibiosolutions.com (S.G.); echarki.zerif@usherbrooke.ca (E.Z.); patrick.mcdonald@usherbrooke.ca (P.P.M.); olga.tatsiy@usherbrooke.ca (O.T.); claude.asselin@usherbrooke.ca (C.A.); gilles.dupuis@usherbrooke.ca (G.D.); denis.gris@usherbooke.ca (D.G.)

**Keywords:** tolerogenic bone marrow-derived dendritic cells (BMDCs), CCAAT/enhancer-binding protein beta (C/EBPβ), Interleukins 10 and 12 (IL-10; IL-12)

## Abstract

Dendritic cells (DCs) play a major role in innate and adaptive immunity and self-immune tolerance. Immunogenic versus tolerogenic DC functions are dictated by their levels of costimulatory molecules and their cytokine expression profile. The transcription factor C/EBPβ regulates the expression of several inflammatory genes in many cell types including macrophages. However, little is known regarding the role of C/EBPβ in tolerogenic versus immunogenic DCs functions. We have previously reported that bone marrow-derived DCs generated with GM-CSF (GM/DCs) acquire the signature of semi-mature tolerogenic IL-10-producing DCs as opposed to immunogenic DCs generated with GM-CSF and IL-4 (IL-4/DCs). Here, we show that tolerogenic GM/DCs exhibit higher levels of phosphorylation and enhanced DNA binding activity of C/EBPβ and CREB than immunogenic IL-4/DCs. We also show that the p38 MAPK/CREB axis and GSK3 play an important role in regulating C/EBPβ phosphorylation and DNA binding activity. Inhibition of p38 MAPK in GM/DCs resulted in a drastic decrease of C/EBPβ and CREB DNA binding activities, a reduction of their IL-10 production and an increase of their IL-12p70 production, a characteristic of immunogenic IL-4/DCs. We also present evidence that GSK3 inhibition in GM/DCs reduced C/EBPβ DNA binding activity and increased expression of costimulatory molecules in GM/DCs and their production of IL-10. Analysis of GM/DCs of C/EBPβ^−/−^ mice showed that C/EBPβ was essential to maintain the semimature phenotype and the production of IL-10 as well as low CD4^+^ T cell proliferation. Our results highlight the importance of the p38MAPK-C/EBPβ pathway in regulating phenotype and function of tolerogenic GM/DCs.

## 1. Introduction

Dendritic cells (DCs) are potent antigen presenting cells (APCs) that play a crucial role in linking innate and adaptive immunity. They detect microorganisms and induce an antigen-specific immunity against pathogens, and can also control and maintain immune tolerance against self antigens [1]. DCs normally reside in tissues in an immature form (iDCs) but they differentiate into semi-mature (smDCs) or fully mature (mDCs) DCs following activation of maturation signals. These signals can be induced by host-derived inflammatory cytokines (CD40L, TNFα, IL-1, IL-6, and IFNγ), or by microbial products and molecules that stimulate Toll-like receptors [2]. Depending on their state of maturation, DCs exhibit distinct APC functions [3] such as production large amounts of inflammatory cytokines such as TNFα, IL-1, IL-6, and IL-12, or anti-inflammatory cytokines such as IL-10 [4]. IL-12 produced by mDCs drives polarization of T cells toward a Th1 phenotype, whereas IL-10-producing smDCs inhibit Th1 responses and are involved in the differentiation of regulatory T cells (Tregs) [5,6]. We and others have reported that bone marrow-derived smDCs produce high amounts of IL-10 but low amounts of IL-12 and play a critical role in promoting and maintaining non-inflammatory immune T cell responses [5,6,7,8,9,10,11]. These in vitro findings are corroborated by other studies that showed that in vivo treatment with GM-CSF protects from autoimmune diseases such as T1D and thyroiditis by inducing tolerogenic semi-mature splenic myeloid CD8α^−^ DCs that produced less IL-12 but more IL-10 and facilitated Treg expansion [6,12]. Thus, the state of DC maturation and the balance between inflammatory and anti-inflammatory cytokines determine the type of subsequent acquired immune response.

The mechanisms regulating gene expression associated with DCs maturation and function are not fully understood. The DC maturation program involves activation of the transcription factor NF-κB [13,14,15,16] and one or several components of the MAP kinase pathways [15,17,18]. It has been reported that NF-κB is a potent activator of genes encoding costimulatory molecules and pro-inflammatory cytokines during DC maturation [19,20], whereas ERK activation has been associated with promotion of IL-10 production and inhibition of IL-12 production by DCs [21,22]. In connection with these findings, we have reported that inhibition of the MEK1/2-AP-1 pathway reduced the production of IL-10 and enhanced the production of IL-12p70 but had no effect on the phenotype of semi-mature GM/DCs [8]. In addition, it has been reported that ERK was necessary but not sufficient for IL-10 production by smDCs [23]. Consistent with these observations, IL-10 production by TLR-activated DCs was decreased in the presence of ERK inhibitor and by ERK-deficient DCs [24]. Thus, abrogation of ERK activity leads to a reduction, but not abolition of IL-10 expression, suggesting that other pathways may cooperate to IL-10 production by DCs. The transcription factor CCAAT/enhancer binding protein-β (C/EBPβ), a member of the C/EBP family of leucine zipper transcription factors, has been shown to participate in the regulation of many cytokine gene expression associated with inflammatory responses in innate and adaptive immunity [25]. The expression and the activity of C/EBPβ are regulated by inflammatory mediators such as TNFα and LPS [25]. In macrophages, C/EBPβ is involved in induction of gene encoding inflammatory molecules, including IL-12p70, IL-6, and IL-10 [25,26]. C/EBPβ has been also suggested to bind to the IL-10 promoter and to induce its transcription in macrophages and T cells [27,28]. Consistent with these findings, LPS-stimulated macrophages of C/EBPβ-deficient mice produced less IL-12p70, IL-6 and TNFα and did not skew immune response toward the Th1 response [29,30]. Furthermore, a defective expression of IL-12p35 but not IL-12p40 gene expression was observed, thereby explaining the defective expression of bioactive IL-12p70 and impaired Th1 response of C/EBPβ-deficient mice [29]. In contrast to these findings, another study reported comparable expression of these cytokines in C/EBPβ^+/+^ and C/EBPβ^−/−^ LPS-stimulated macrophages [31].

C/EBPβ DNA binding affinity and specificity are regulated through cooperation with other transcription factors such as the p50 subunit of NF-κB, CREB/ATF, AP-1, and SP1 [26]. It has been shown that the interaction between SP1 and C/EBPβ cooperatively activated IL-10 gene expression in LPS-stimulated macrophages [28] and that the CREB-C/EBPβ cascade was essential for induction of the type 2 macrophages anti-inflammatory cytokines such as IL10 [32,33,34,35,36,37]. CREB activity is mainly regulated through its phosphorylation at serine residue 133 by kinases p38 MAPK [38] and by GSK-3 and, at serine residue 129, by GSK-3 [39]. In this connection, it has been shown that C/EBPβ phosphorylation by GSK3 and by MAPK induces a conformational change that increases its DNA binding activity and transcriptional activation [40]. Interestingly, GSK3 inhibition augments CREB DNA binding, reduces IL-12 production but increases IL-10 production in response to a variety of TLR agonists [39]. Despite the well-documented importance of CREB-C/EBPβ axis in macrophages, its role in regulating phenotype and anti-inflammatory IL-10 and proinflammatory IL-12p70 production by tolerogenic versus immunogenic DCs is not fully investigated. In the present study, we present evidence that C/EBPβ and CREB are involved in regulating the balance between IL-10 and IL-12p70 production and in limiting the expression of costimulatory molecules in tolerogenic GM/DCs thereby limiting T cell proliferation. Furthermore, our study defines the molecular pathways that regulate the p38 MAPK-C/EBPβ pathway in tolerogenic GM/DCs.

## 2. Materials and Methods

### 2.1. Mice

C57BL/6, BALB/c, and NOD mice were purchased from Taconic (Hudson, NY, USA). NOD-BDC2.5 and NOD.SCID mice were purchased from the Jackson Laboratory (Bar Harbor, ME, USA). 129SV-C57BL/6 C/EBPβ^−/−^ mice were obtained from Dr. Claude Asselin (Université de Sherbrooke) with the agreement of Dr. Peter F Johnson (National Cancer Institute, Frederick, MD, USA). All mice were housed under pathogen-free conditions, in accordance with the guidelines of the Institutional Animal Care Committee of the University of Sherbrooke (Protocols 93-14 and 208-09).

### 2.2. Preparation of BMDCs

Bone marrow-derived DCs were generated as described [41] with few modifications. Briefly, bone marrow cells were obtained by flushing femurs and tibia and cultured at 10 × 10^6^ cells/10 mL of RPMI 1640 medium containing 10% FBS, glutamine, sodium pyruvate, penicillin/streptomycin, β-mercaptoethanol (50 μM), and 5 ng/mL of GM-CSF alone or with a combination of GM-CSF and 4.5 ng/mL of IL-4 (Cedarlane, Burlington, ON, Canada) in Petri dishes (UltiDent scientific, St. Laurent, QC, Canada). At day 3, 10 mL of fresh medium supplemented with GM-CSF (5 ng/mL) alone or in combination with IL-4 (4.5 ng/mL) was added to the culture. At day 5, half of the medium was removed and replaced with fresh medium supplemented with GM-CSF (5 ng/mL) alone or in combination with IL-4 (4.5 ng/mL). At day 7, non-adherent cells were gently harvested, pooled, and left unstimulated or exposed to LPS (Sigma-Aldrich, St. Louis, MO, USA; 1 μg/mL) for 24 h or 48 h. More than 90% of these non-adherent cells expressed moderate to high levels of CD11c/MHC-Class II and less than 10% were Gr1 positive. Bone marrow-derived IL-10/DCs were generated with GM-CSF (5 ng/mL), IL-4 (4.5 ng/mL), and IL-10 (10 ng/mL) (Cedarlane) following the same protocol.

### 2.3. Flow Cytometry and Antibodies

Anti-CD11c (clone HL3), anti-CD86 (clone GL1), anti-CD40 (Clone 1C10), and anti-CD80 (clone 16-10A1) were purchased from BD Biosciences (Mississauga, ON, Canada). For FACS analysis, DCs were washed with PBS and stained with an anti-CD11c-FITC mAb in combination with anti-CD80-PE-Cy5, anti-CD86-PE-Cy5, or anti-CD40-PE-Cy5 mAbs. Conjugated matched isotypes (Armenian hamster IgG2 or Rat IgG2a) were used as negative controls. Cells were acquired using or FACSCalibur flow cytometer (BD Biosciences) or FACSCanto Instrument (BD Biosciences, CA, USA), and analyzed using the CellQuest Pro (BD Biosciences) or FlowJo (Tree Star, Ashland, OR, USA) software.

### 2.4. Proliferation Assays and Cytokine Quantification

Purified CD4^+^ T cells were cultured with in the presence of anti-CD3 and anti-CD28 Abs in the with the addition of GM/DCs or IL-4/DCs for three days at 37 °C. At day 2 of culture, supernatants were collected for cytokine quantification using ELISA kits (R&D Systems, Minneapolis, MN, USA). Cells were pulsed with 1 μCi of [^3^H] thymidine/well during the last 18 h of culture and radioactivity was counted. For antigen-specific CD4^+^ T cell proliferation and cytokine production, CD4^+^CD25^−^ T cells (2 × 10^4^ cells/well) purified from DBC2.5-NOD mice were incubated with NOD GM/DCs (5 × 10^3^ cells/well) that have been pre-incubated with the p38 MAPK inhibitor or vehicle and pulsed with the BDC2.5 mimotope 1040-31 peptide (0.5 μg/mL) (Cederlane, Burlington, ON, Canada). IFNγ was quantified in the supernatants after 48 h of culture using ELISA and proliferation was determined using the thymidine incorporation assay described above.

### 2.5. Protein Kinase Inhibition

BMDCs were pre-incubated with the p38 MAPK inhibitor SB203580 (10 μM), the GSK-3 inhibitors SB216763 (10 μM) or LiCl (10 mM) or, with DMSO (vehicle) for 1 h prior to cell stimulation with LPS (1 μg/mL) as described before [8].

### 2.6. Nuclear Extract Preparation

BMDCs were recovered by centrifugation and cell pellets were re-suspended in cold lysis buffer (10 mM Tris base, pH 7.4, containing 10 mM NaCl, 3 mM MgCl_2_, 0.5 mM EGTA, 0.5 mM EDTA, 0.5 mM DTT, and protease and phosphatase inhibitors). After a 10 min of incubation on ice, an equal volume of the lysis buffer containing 0.2% NP-40 was added and the extracts were centrifuged at 1500× *g* for 5 min at 4 °C. The cytoplasmic fractions were recovered in the supernatants. The nuclear pellets were re-suspended in lysis buffer containing 10% glycerol and NaCl was added to a final concentration of 400 mM. After 20 min incubation on ice, the samples were centrifuged at 13,000× *g* (15 min, 4 °C) and supernatants were collected and used as nuclear extracts.

### 2.7. EMSA and Supershift Assays

EMSAs were conducted as described [42]. Briefly, nuclear extracts were added to 18 µL of total volume of binding buffer (20 mM Tris Base pH 7.5, 50 mM KCl, 1 mM EDTA, 1 mM DTT, 0.1% NP40, 5% glycerol) containing 6 µg of acetylated BSA and 0.8 µg of poly dI-dC. Then, 10 fmoles of ^32^P-end-labeled oligonucleotide were added to the binding reaction, which was further incubated for 15 min at room temperature. In the case of supershift experiments, binding reactions were performed in the presence of appropriate antibodies for 30 min at 4 °C, before addition of labeled oligonucleotides. Samples were electrophoresed on 5.5% native polyacrylamide gels in Tris-Borate EDTA (TBE) 0.5X at 4 °C. Dried gels were exposed to autoradiography film at −80 °C. Oligonucleotides used were 5′-AGAGATTGCCTGACGTCAGAGAGCTAG-3′ in the case of CREB and 5′-TGCAGATTGCGCAATCTGCA-3′ in the case of C/EBP. Antibodies used for supershift assays were anti-C/EBPα (sc-61X), anti-C/EBPβ (sc-105X), anti-C/EBPδ (sc-151X), anti-C/EBPε (sc-158X), and anti-C/EBPγ (sc-7658X) (Santa Cruz Biotechnology, Santa Cruz, CA, USA).

### 2.8. Western Blots

BMDCs were harvested, washed in cold PBS and resuspended in lysis buffer containing Tris base 50 mM, NaCl 0.15 M, DTT 1 mM, Triton X-100 1% (v/v) and a cocktail of protease and phosphatase inhibitors. Cell lysates were fractionated on 10% SDS-PAGE gels, transferred to nitrocellulose membrane (Hybond-ECL Amersham Biosciences, Baie d’Urfé, QC, Canada) and incubated overnight with primary antibodies directed against pC/EBPβ, C/EBPβ, pCREB (Ser133), CREB, β-actin (Cell Signaling Technology Inc., Danvers, MA, USA), or pCREB (Ser129) (Santa Cruz Biotechnology, Santa Cruz, CA, USA). Blots were then incubated with appropriate secondary antibodies and revealed by enhanced chemiluminescence (GE Health Care, Baie d’Urfé, QC, Canada).

### 2.9. Measurements of Cytokine Production

BMDCs were plated in 96-well culture plates at a density of 1 × 10^6^ cells/mL in the absence or the presence of LPS (1 μg/mL) for 24 h. IL-10, IL-12p70, and IL-6 production was quantified in the supernatants using ELISA kits, according to manufacturer’s instructions (R&D Systems).

### 2.10. Real-Time PCR Analysis

Total RNA was extracted from 5 × 10^6^ BMDCs using Trizol (Invitrogen, Burlington, ON, Canada) and 1 µg of the resulting RNA was reverse transcribed using Superscript II (Invitrogen, Burlington, ON, Canada) and OligodT (Promega, Madison, WI, USA). Duplicate real-time PCR reactions for each sample were performed in a volume of 25 µL containing 10 ng of cDNA and 1 µm of each forward and reverse primers (Supplementary Table S1), using a Quantitect SYBR Green qPCR kit (Qiagen, Montreal, Quebec, Canada) in a Rotorgene 3000 instrument (Corbett Research, Sydney, Australia). Reaction conditions were as follows: 95 °C for 5 min, followed by 35 cycles (94 °C for 30 s, 60 °C for 45 s, 72 °C for 60 s in the case of IL-12p35 and IL-12p40 or 94 °C for 30 s, 64 °C for 45 s, 72 °C for 60 s in the case of IL-10 and TGF-β. Amplification plots were generated using the Rotorgene Amplification software v6.0 (Corbett Research) and fold increases were calculated using the 2^−ΔΔCt^ method and normalized using β-actin expression.

### 2.11. Statistics

Data were analyzed using the GraphPad Prism 6.0 software (GraphPad, San Diego, CA, USA) and are shown as the mean ± SEM. The Mann–Whitney test was used to detect differences between two groups of nonparametric unpaired samples. When more than two groups were compared, one-way ANOVA with post hoc Bonferroni’s test were done. Differences were considered to be statistically significant for *p* < 0.05.

## 3. Results

### 3.1. C/EBPβ DNA Binding Activity in GM/DCs and in IL-4/DCs

**Figure 1:**
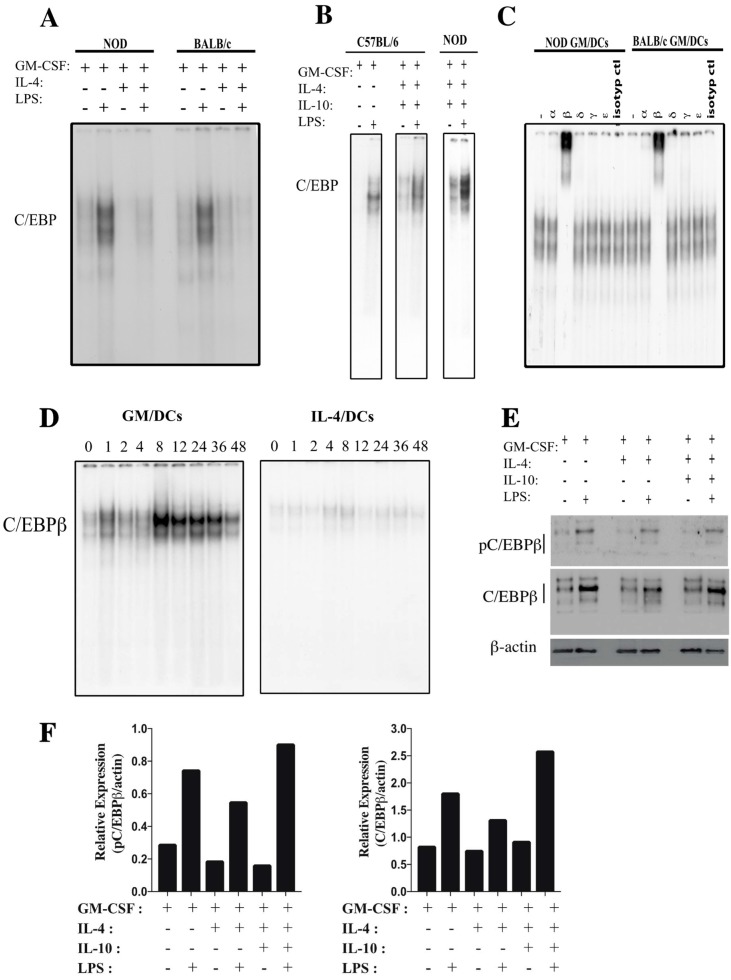
Increased C/EBPβ expression and DNA binding activity in LPS-stimulated GM/DCs. (**A**) Bone marrow-derived GM/DCs and IL-4/DCs were generated from NOD and BALB/c mice and left unstimulated or stimulated with LPS (1 μg/mL for 48 h). Nuclear extracts were subjected to EMSA assays to assess C/EBP binding activity. (**B**) Bone marrow-derived GM/DCs and IL-10/DCs (generated with GM-CSF + IL-4 + IL-10) from C57BL/6 and NOD mice were left unstimulated or stimulated with LPS (1 μg/mL for 48 h). Nuclear extracts were prepared and C/EBP binding activity was determined by EMSA assays (**C**) Supershift analyses were performed with nuclear extracts obtained from LPS-stimulated GM/DCs of NOD and BALB/c mice, as described in the Material and Methods section of the body of the text. (**D**) C/EBPβ DNA binding activity was monitored by EMSA, using nuclear extracts from LPS-stimulated GM/DCs and IL-4/DCs from NOD mice at the indicated times. (**E**) Western blot analysis of pC/EBPβ (Thr188) and C/EBPβ levels in cell lysates from LPS stimulated GM/DCs and IL-4/DCs. (**F**) Quantitative analysis of data in Figure 1E normalized to loading control (β-actin). Data are representative of a minimum of 2–3 independent experiments.

We have reported that GM/DCs of NOD mice possess properties of tolerogenic DCs such as exhibition of a semi-mature phenotype and production of high amounts of IL-10 and low amounts of IL-12 [8]. We also have reported that the ERK1/2 MAP kinase was important but not sufficient to regulate the IL-10/IL-12p70 balance but was not involved in regulating DC maturation. C/EBPβ has been also shown to be a critical regulator of the expression of IL-10 and IL-12p35 in macrophages [29,43] but its role in DCs maturation status and IL-10 and IL-12p70 production has not been fully investigated. In a first series of experiments, we examined (EMSA) C/EBP DNA binding activity in nuclear extracts prepared from GM/DCs and IL-4/DCs of NOD and BALB/c mice. Results showed a weak C/EBP DNA binding activity in unstimulated GM/DCs and IL-4/DCs of both strains of mice (Figure 1A). However, following LPS-stimulation, C/EBP DNA binding activity was strongly increased in GM/DCs but not in IL-4/DCs from both strains of mice (Figure 1A). Similar results were obtained with bone morrow-derived DCs of C57BL/6 mice (Figure 1B). To investigate whether the strong C/EBP DNA binding activity observed in the case of GM/DCs was associated with tolerogenic potential of DCs, C/EBP DNA binding activity was analyzed in IL-10/DCs (generated in the presence of GM-CSF, IL-4, and IL-10) of NOD and C57BL/6 mice that have been reported to possess tolerogenic properties [44,45,46,47]. Interestingly, strong C/EBP DNA binding activity was also observed in LPS-stimulated IL-10/DCs of NOD and C57BL/6 mice (Figure 1B). These data indicated that increased C/EBP binding activity in tolerogenic GM/DCs and IL-10/DCs of NOD mice was congruent with findings in BALB/c or C57BL/6 mice, suggesting that these results held true across these three strains of mice. To determine which one of the c/EBP isoforms bound to C/EBP consensus sequence, supershift assays using nuclear extracts of LPS-stimulated GM/DCs were performed. Results revealed that only the anti-C/EBPβ antibody shifted the DNA binding complex in GM/DCs’ nuclear extracts prepared from NOD and BALB/c mice (Figure 1C). Furthermore, a time course study (Figure 1D) showed that C/EBPβ DNA binding activity in LPS-stimulated GM/DCs was increased at 1 h post-stimulation, peaked at 8 h, and progressively decreased thereafter. However, binding activity remained higher at all time points compared to the very weak DNA binding activity in IL-4/DCs. Western blot analysis showed that the levels of CEBPβ and phosphorylated CEBPβ were higher in LPS-stimulated GM/DCs and IL-10/DCs than in LPS-stimulated IL-4/DCs (Figure 1E,F). Moreover, C/EBPβ protein was found in the nuclear extracts and its expression was higher in GM/DCs than in IL-4/DCs (Supplementary Figure S1). Together, these results showed an increased C/EBPβ phosphorylation that correlated with enhanced DNA binding activity in LPS-stimulated tolerogenic GM/DCs but not in immunogenic LPS-stimulated IL-4/DCs.

### 3.2. Effect of p38 MAPK and GSK3 Inhibitors on C/EBPβ and CREB Phosphorylation and DNA Binding Activity in GM/DCs

**Figure 2:**
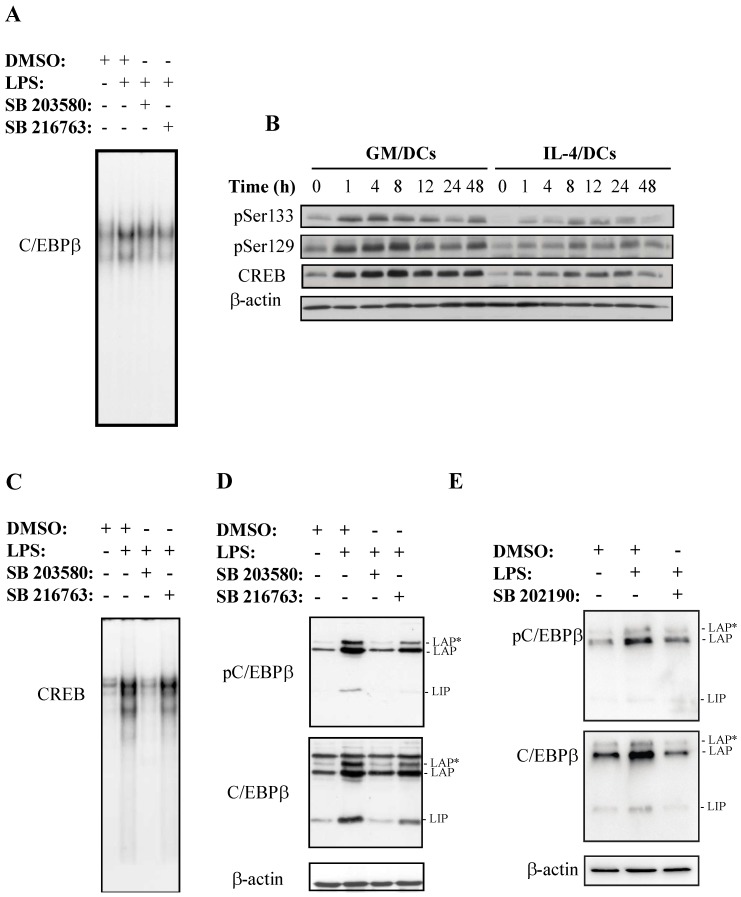
Role of p38 MAPK/CREB and GSK3b/CREB axis in the activation and DNA binding activity of C/EBPβ in GM/DCs. Bone marrow-derived GM/DCs were preincubated with inhibitors of p38 MAPK (SB203580, 10 μM), p38 MAPK (SB202190, 1 μM), GSK3 (SB 216763, 10 μM), or vehicle (DMSO) for 1 h before stimulation with LPS (1 μg/mL for 8 h). (**A**) C/EBPβ DNA binding activity in nuclear extracts were analyzed by EMSA. (**B**) Western blot analysis of the levels of phosphorylated (Ser129, Ser133) and total CREB in whole cell lysates at the indicated times (hours) after LPS stimulation (1 μg/mL). (**C**) CREB DNA binding activity in nuclear extracts were analyzed by EMSA. (**D**,**E**) Western blot analysis of the levels of phosphorylated (Thr188) and total C/EBPβ in whole cell lysates after 8 h of LPS stimulation (1 μg/mL). Data are representative of two independent experiments.

It has been reported that phosphorylation of C/EBPβ by MAPK and, subsequently, by GSK3β leads to the acquisition of DNA binding activity [48]. To determine the contribution of each kinase on C/EBPβ DNA binding activity, GM/DCs were treated with the p38 MAPK inhibitor SB203580 or with the GSK3 inhibitor SB216763 prior to stimulation with LPS. Results of EMSA experiments showed that both inhibitors reduced C/EBPβ DNA binding activity (Figure 2A). The downstream targets of p38 MAPK include a wide array of cytoplasmic and nuclear factors including CREB, which is known to induce C/EBPβ transcription following its phosphorylation on serine residues [32,37]. Western blot analysis clearly showed a rapid and intense CREB phosphorylation at Ser133 and Ser129 in GM/DCs, as compared to low levels of CREB phosphorylation and expression in IL-4/DCs (Figure 2B). Next, we investigated whether the p38 MAPK inhibitor SB203580 would affect CREB DNA binding activity in GM/DCs. Results showed that the strong CREB DNA binding activity observed in LPS-stimulated GM/DCs was drastically reduced in the presence of the p38 MAPK inhibitor SB203580 whereas the GSK3 inhibitor SB216763 had no effect (Figure 2C). Consistent with these observations, a drastic decrease in the levels of protein and phosphorylation levels at Thr188 of C/EBPβ was observed in the presence of the p38 MAPK inhibitors SB203580 (Figure 2D) and SB202190 (Figure 2E). In contrast, addition of the GSK-3β inhibitor led to a slight decrease of C/EBPβ phosphorylation and C/EBPβ protein levels, particularly the LIP isoform (Figure 2D). These data indicated that both the p38 MAPK-CREB axis and GSK3β play a critical role in inducing C/EBPβ phosphorylation and DNA binding activity in tolerogenic GM/DCs.

### 3.3. Functional Impact of Inhibition of C/EBPβ and CREB DNA Binding on Cytokine Production and Phenotype of Tolerogenic GM/DCs

**Figure 3:**
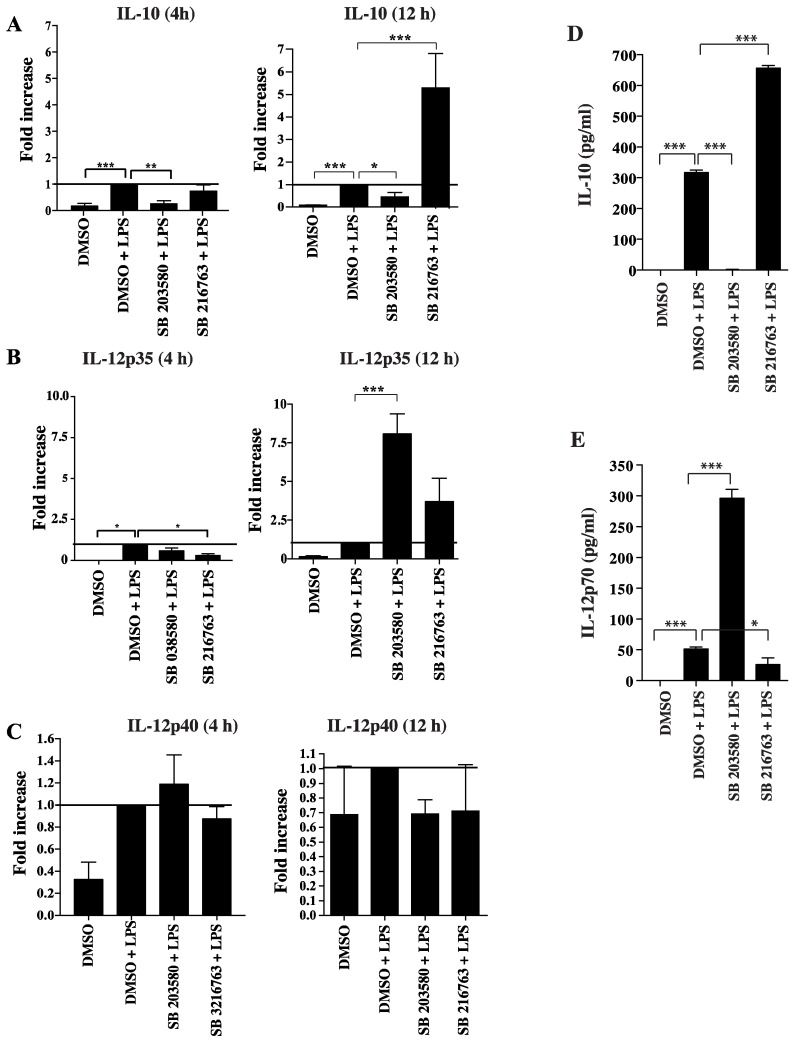
Role of p38 MAPK/CREB and GSK3β/CREB axis in regulating IL-10 and IL-12 in GM/DCs. Bone marrow-derived GM/DCs were preincubated for 1 h in the presence of a p38 MAPK (SB203580, 10 µM) or a GSK3 (SB216763, 10 µM) inhibitor or, vehicle (DMSO) before being exposed to LPS (1 μg/mL for 4 h or 12 h). Total RNA was isolated and the expression of IL-10 (**A**), IL-12p35 (**B**) and IL-12p40 (**C**) was determined by real-time PCR. Fold increases are calculated with reference to vehicle (DMSO) + LPS conditions. Data are representative of five or six independent experiments. In the case of cytokine release essays, GM/DCs were preincubated for 1 h with SB203580 (10 µM), SB216763 (10 µM) or, with vehicle (DMSO), before being exposed to LPS (1 μg/mL for 24 h). IL-10 (**D**) and IL-12p70 (**E**) production was quantified in cell supernatants by ELISA. Data are representative of three independent experiments. Results are shown as the mean ± SEM. The asterisks (*) correspond to *p <* 0.05 (*), *p <* 0.01 (**), and *p <* 0.001 (***), one-way ANOVA with post hoc Bonferroni’s test.

**Figure 4:**
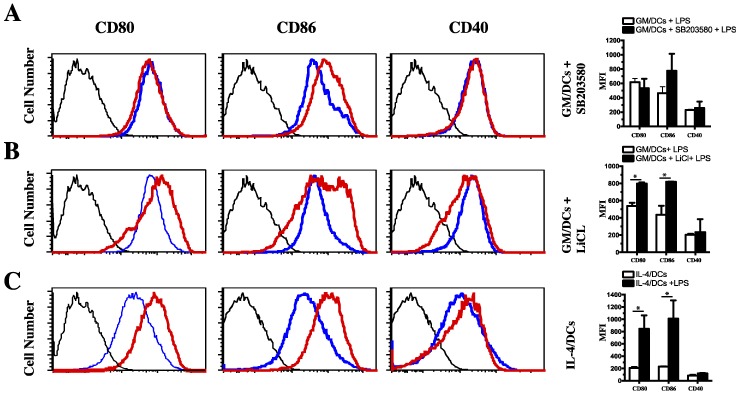
Role of p38 MAPK/CREB and GSK3b/CREB axis in regulating GM/DCs maturation status. Bone marrow-derived GM/DCs were preincubated with vehicle (DMSO, Blue thin line) or (**A**) p38 MAPK inhibitor (SB203580. 10 µM, red line) or (**B**) LiCl (10 mM, red line) before being exposed to with LPS (1 μg/mL for 48 h). Cells were harvested and the expression of CD80, CD86, and CD40 molecules was analyzed by gating on CD11c^+^ cells using flow cytometry. (**C**) Bone marrow-derived IL-4/DC were left unstimulated (blue line) or were exposed to LPS (1 μg/mL for 48 h) (red line) and analyzed by flow cytometry. For all the panels, isotype controls are shown (dashed thin line). Expression levels of CD80, CD86, and CD40 cell surface markers are illustrated as mean fluorescence intensities (MFIs) are shown at right-hand sides of panels. Data are representative of two to four independent experiments. * *p* < 0.05, nonparametric unpaired Mann–Whitney test.

C/EBPβ is involved in the production of IL-10 [43] and IL-12 by regulating IL-12p35 and IL-12p40 gene expression [29] in macrophages. We therefore investigated whether inhibition of C/EBPβ binding activity in LPS-stimulated GM/DCs would affect gene expression and production of IL-10 and IL-12p70 cytokines. In agreement with our previous report [8], results showed that LPS induced a significant increase in mRNA expression and secretion of IL-10, whereas it only promoted a modest induction of IL-12p35 expression and IL-12p70 secretion (Figure 3). Interestingly, pretreatment with the p38 MAPK inhibitor SB203580 significantly (*p* < 0.05) reduced IL-10 gene expression and secretion (Figure 3A,D) whereas it markedly (*p* < 0.001) enhanced IL-12p35 gene expression and IL-12p70 secretion (Figure 3B,E). In contrast, a pretreatment with the GSK3 inhibitor SB216763 significantly (*p* < 0.001) enhanced IL-10 mRNA expression and protein secretion (Figure 3A,D). The addition of SB216763 led low IL-12p35 mRNA expression at 4 h followed by enhanced IL-12p35 mRNA expression at 12 h of LPS stimulation, although to a lesser extent than p38 MAPK inhibition (Figure 3B). In contrast, SB216763 significantly reduced IL-12p70 production, suggesting that GSK3 inhibition results in downregulation of IL-12p70 production through inhibition of IL-12p35 translation (Figure 3E). No significant changes were observed in IL-12p40 mRNA expression in the presence of SB203580 or SB216763 inhibitors (Figure 3C). Treatment with LiCl, another GSK3 inhibitor, resulted in a similar switch in the profile of IL-10 and IL-12p70 secretion, as observed with SB216763 inhibitor (Supplementary Figure S2). Taken together, these results suggested that p38 MAPK and GSK3 kinases are involved in the regulation of the CREB/C/EBPβ axis, which appears to be important in fine-tuning the balance between IL-10 and IL-12p70 production in GM/DCs. We next analyzed the expression of costimulatory molecules CD80, CD86, and CD40 to determine whether the production of IL-10 and IL-12p70 by GM/DCs incubated in the presence of p38 MAPK and GSK3 inhibitors was accompanied by an increased maturation of GM/DCs upon LPS stimulation. Results of FACS analysis showed that the use of the p38 MAPK inhibitor SB203580 resulted in a weak increase in expression of CD86 but not CD80 or CD40 (Figure 4A). In marked contrast, the LiCl-dependent inhibition of GSK3 strongly enhanced the expression of CD80 and CD86 but not CD40 (Figure 4B). This mature phenotype of LPS-stimulated GM/DCs acquired in the presence of GSK3 inhibitor was similar to that of mature LPS-stimulated IL-4/DCs generated in the presence of GM-CSF and IL-4 (Figure 4C). Together, these data strongly suggested that the p38MAPK/CREB/C/EBPβ axis regulates IL-10 and IL-12 production by LPS-stimulated GM/DCs and further suggested that the p38MAPK/GSK3β-C/EBPβ axis was involved in regulating GM/DCs phenotype and function.

### 3.4. Effect of C/EBPβ Deficiency on the Phenotype of GM/DCs

**Figure 5:**
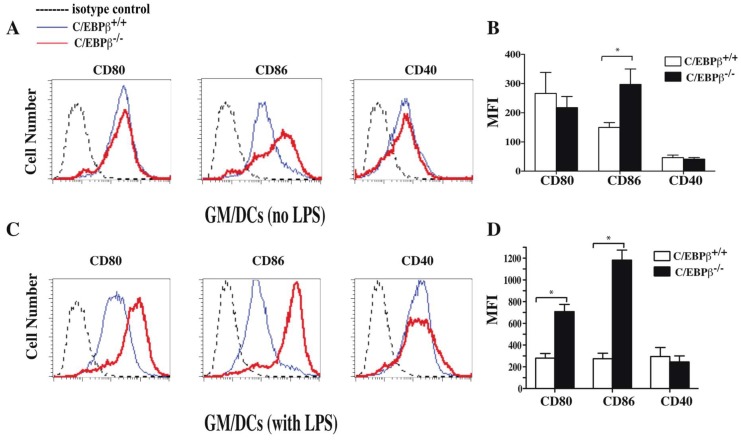
Effect of C/EBPβ deficiency on the maturation status of GM/DCs. Bone marrow-derived GM/DCs generated from C/EBPβ^+/+^ mice (Blue line) and C/EBPβ^−/−^ mice (red line), were left unstimulated (**A**) or exposed to LPS (1 µg/mL for 48 h) (**C**) and were stained and analyzed for CD80, CD86, and CD40 expression on CD11c^+^ cells using flow cytometry. Dashed thin lines represent labeling with isotype control antibodies. (**B** and **D**) Expression levels of CD80, CD86, and CD40 cell surface markers on unstimulated (**B**) and LPS exposed (**D**) GM/DCs are illustrated as mean fluorescence intensities (MFIs). The results are representative of three independent experiments. * *p* < 0.05, nonparametric unpaired Mann—Whitney test.

To further evaluate the role of C/EBPβ in regulating DCs maturation status, GM/DCs were derived from bone marrow of 129SV/C57BL6 C/EBPβ^−/−^ (C/EBPβ^−/−^) mice and their littermate controls 129SV/C57BL6 C/EBPβ^+/+^ (C/EBPβ^+/+^) and were stimulated or not with LPS. Results of FACS analysis showed that unstimulated GM/DCs from both strains of mice expressed low levels of CD80 and CD40 whereas the level of CD86 was higher in GM/DCs from C/EBPβ deficient mice (Figure 5A,B). In agreement with our previous report [8], LPS-stimulated GM/DCs of C/EBPβ^+/+^ mice displayed a semi-mature phenotype characterized by a weak increase of expression of CD80 and CD86 markers (Figure 5A,D). Interestingly, LPS-stimulated GM/DCs derived from C/EBPβ^−/−^ mice displayed a fully mature phenotype, as indicated by enhanced expression of CD80 and CD86 compared to GM/DCs of C/EBPβ^+/+^ mice (Figure 5C,D). In addition, the levels of CD80 and CD86 in LPS-stimulated GM/DCs of C/EBPβ^−/−^ mice were similar to those expressed by LPS-stimulated IL-4/DCs derived from C/EBPβ^+/+^ mice (Figure 4C). These data suggest that C/EBPβ plays a determining role in limiting GM/DCs maturation.

### 3.5. Role of C/EBPβ in Regulating GM/DCs Function

**Figure 6:**
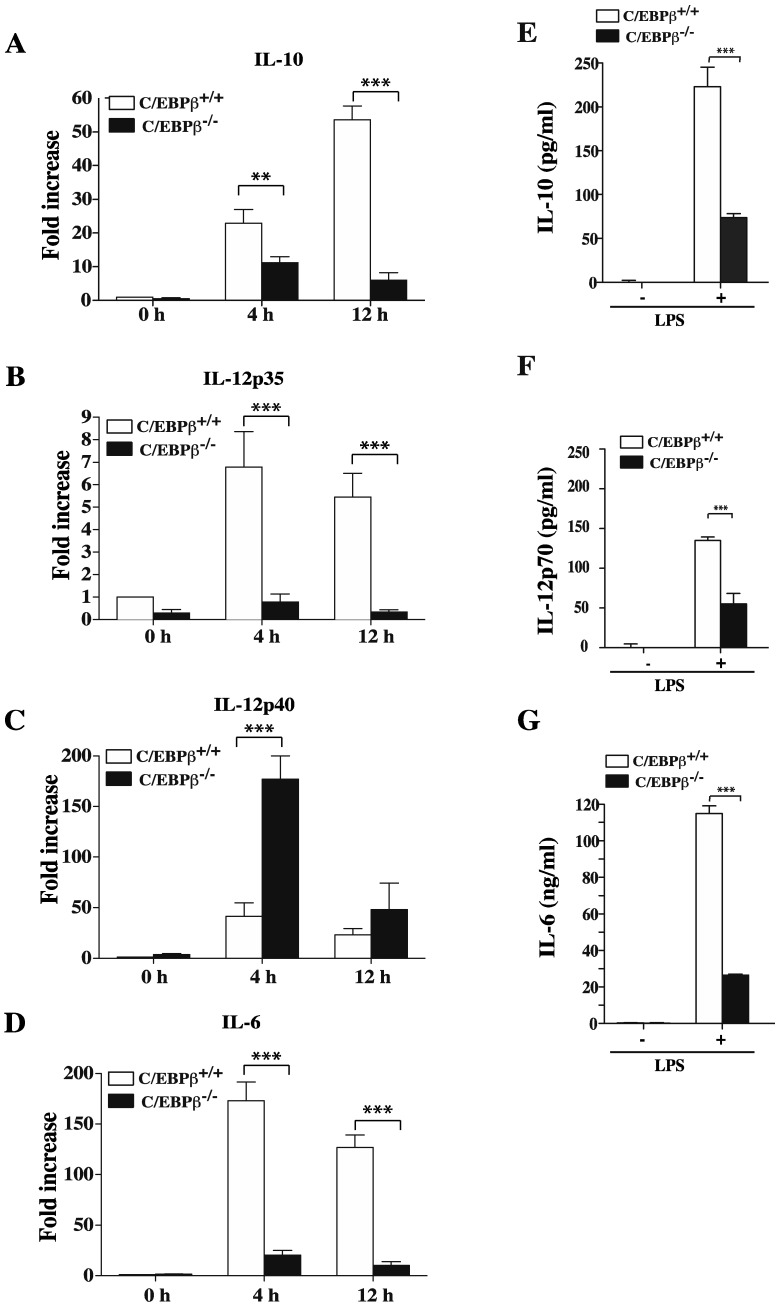
Effect of C/EBPβ deficiency on gene expression and production of IL-10 and IL-12 by GM/DCs. Bone marrow-derived GM/DCs generated from C/EBPβ^−/−^ or C/EBPβ^+/+^ mice were left unstimulated or were stimulated with 1 µg/mL of LPS for 4 h and 12 h. The expression of IL-10 (**A**), IL12p35 (**B**), IL-12p40 (**C**), and IL-6 (**D**) was determined by real-time PCR. Data are shown as fold increases with reference to unstimulated GM/DCs of C/EBPβ^+/+^ (t = 0) and are representative of four independent experiments. For cytokines release, bone marrow-derived GM/DCs generated from C/EBPβ^−/−^ or C/EBPβ^+/+^ mice were left unstimulated or stimulated with LPS (1 µg/mL for 24 h). Cell supernatants were collected and IL-10 (**E**), IL-12p70 (**F**), and IL-6 (**G**) production was analyzed by ELISA. Data are representative of three independent experiments and are shown as the mean ± SEM. The asterisks (*) correspond to *p* < 0.01 (**) and *p <* 0.001 (***), one-way ANOVA with post hoc Bonferroni’s test.

**Figure 7:**
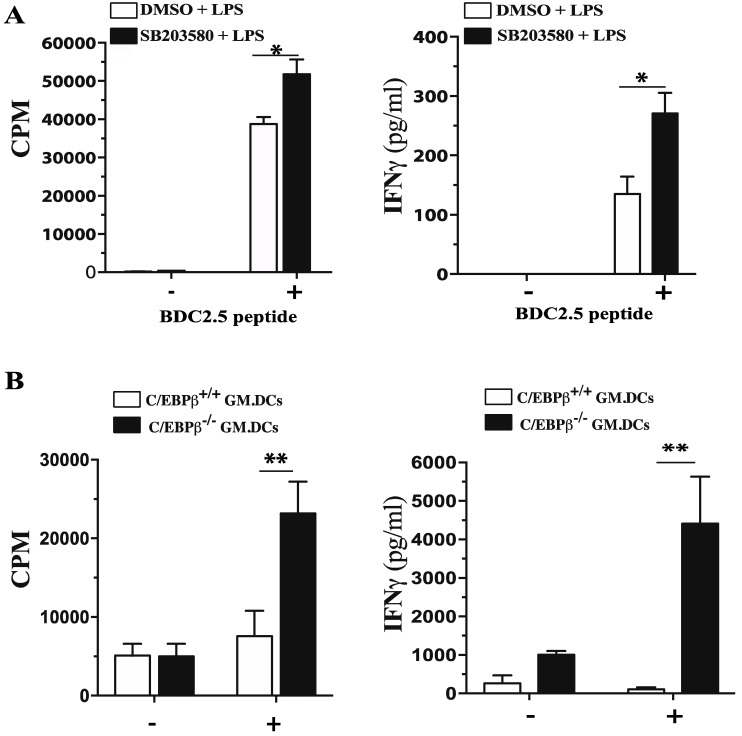
Elevated CD4^+^ T cell proliferation and IFNγ production induced by the p38 MAPK inhibitor-treated or C/EBPβ-deficient GM/DCs. (**A**) Proliferative response and IFNγ secretion by autoreactive BDC2.5-CD4^+^ T cells that had been cultured with GM/DCs treated with p38 MAPK inhibitor SB203580 or with vehicle (DMSO) and pulsed or not with BDC2.5-peptide. (**B**) Proliferative response and IFNγ secretion by naïve CD4^+^ T cells purified from C/EBPβ^+/+^ littermate control mice that have been activated with anti-CD3 and anti-CD28 Abs in the presence of C/EBPβ^+/+^ or C/EBPβ^−/−^ GM/DCs before (−) and after (+) LPS stimulation. Quantification of IFNγ released in the supernatant. Data are representative of three independent experiments and are shown as the mean ± SD. The asterisks (*) correspond to *p* < 0.01 (**), one-way ANOVA with post hoc Bonferroni’s test.

We next sought to determine the consequence of C/EBPβ deficiency on mRNA levels and secreted IL-10 and IL-12 in GM/DCs. Results showed that LPS-stimulated GM/DCs of C/EBPβ^+/+^ mice expressed more IL-10 mRNA transcripts than IL-12p35 mRNA transcripts (Figure 6). The expression levels of both transcripts were dramatically reduced in LPS-stimulated GM/DCs derived from C/EBPβ^−/−^ mice (Figure 6A,B). Levels of IL-12p40 mRNA were transiently increased in GM/DCs from C/EBPβ^−/−^ mice (Figure 6C). In agreement with the reported impaired expression of IL-6 in C/EBPβ-deficient macrophages [29], our data showed a significant decrease of IL-6 mRNA in GM/DCs of C/EBPβ^−/−^ mice as compared to GM/DCs of C/EBPβ^+/+^ mice (Figure 6D). Analysis of secreted cytokines showed that, following 24 h of LPS stimulation, GM/DCs of C/EBPβ^−/−^ mice produced lower amounts of IL-10 than GM/DCs from C/EBPβ^+/+^ mice (Figure 6E). Furthermore, GM/DCs of C/EBPβ^−/−^ mice produced significantly lower amounts of IL-12p70 than GM/DCs of C/EBPβ^+/+^ mice (Figure 6F). Similarly, IL-6 production by GM/DCs of C/EBPβ^−/−^ mice was drastically reduced as compared to GM/DCs of C/EBPβ^+/+^ mice (Figure 6G). Two groups have recently reported that, C/EBPβ plays an essential role in limiting proliferation of CD4^+^ T cells and IFNγ production in the case of lymphoma-educated DCs and Gr1+CD11b MDSC [49,50]. These findings led us to investigate the impact of C/EBPβ on the capacity of tolerogenic GM/DCs to induce CD4^+^ T cells proliferation as well as IFNγ production. Results showed islet-specific BDC-2.5 CD4^+^ T cell proliferation and IFNγ production were significantly increased when cultured in the presence of peptide-pulsed LPS-stimulated GM/DCs that has been pretreated with the p38MAPK inhibitor SB203580 as compared to BDC2.5 CD4^+^ T cells cultured with peptide-pulsed LPS-stimulated GM/DCs pretreated with vehicle (Figure 7A). Similarly, CD4^+^ T cell proliferation and IFNγ production were significantly increased in the presence of LPS-stimulated C/EBPβ^−/−^ GM/DCs as compared to the absence of proliferation and IFNγ production by CD4^+^ T cells co-cultured in the presence of LPS-stimulated GM/DCs (Figure 7B). These data were presented as evidence that C/EBPβ was an important transcription factor that controlled not only IL-10 and IL-12 gene and protein expression but that it also influenced the capacity of GM/DCs to induce CD4^+^ T proliferation and IFNγ^+^ effector phenotype. Overall, these results suggest that c/EBPβ is a transcription factor that plays an essential role in establishing a GM/DCs tolerogenic program.

## 4. Discussion

We have previously reported that in vitro bone marrow-derived GM/DCs exhibit a signature of tolerogenic semi-mature IL-10-producing DCs whereas IL-4/DCs exhibit the functional properties and phenotype of fully mature immunogenic IL-12-producing DCs [8]. We have also reported that inhibition of the MEK1/2-AP-1 pathway reduced production of IL-10 and enhanced production of IL-12p70 but had no effect on the phenotype of semi-mature GM/DCs [8]. The transcription factor C/EBPβ has been extensively studied with respect to regulation of IL-10/IL-12 balance in macrophages, but its role in phenotype and function of tolerogenic DC remains to be elucidated. Results reported here showed that semi-mature phenotype and IL-10/IL12 production by tolerogenic GM/DCs were under the control of the p38 MAPK-C/EBPβ axis and that the expression of C/EBPβ was necessary to maintain low levels of expression of CD80/CD86 and high production of IL-10 by tolerogenic GM/DCs as well as their reduced capacity to induce CD4^+^ T cells proliferation and IFNγ production.

DCs coordinate immune response because they play a critical role in induction of immunity and triggering T cell tolerance. It is well established that the maturation stage and nature of cytokines produced by DCs are important determinants of their tolerogenic as opposed to their immunogenic functions. Fully matured DCs potently induce effector T cell responses because of an increased expression of costimulatory molecules such as CD80 and CD86 and pro-inflammatory cytokines. In contrast, low or intermediate expression of maturation markers as well as IL-10 secretion are the characteristics of tolerogenic DCs that are important events in the induction and maintenance of immune tolerance. Of interest, DCs play a critical role in the initiation and progression of autoimmune diseases including type 1 diabetes. We and others have shown that BMDCs generated from NOD mice display a more mature phenotype, produce more proinflammatory cytokines (such IL-12p70) but less anti-inflammatory cytokines (such IL-10) and promote pro-inflammatory immune response [8,51,52]. We have also reported that splenic DCs purified from GM-CSF-treated NOD mice as well as bone marrow-derived GM/DCs exhibit similar properties of semi-mature tolerogenic DCs [6,8]. However, the molecular mechanisms that govern tolerogenic properties of these DCs are not fully understood. Data presented here showed that C/EBPβ was highly activated in tolerogenic GM/DCs following LPS stimulation but not in immunogenic IL-4/DCs. Furthermore, phosphorylated C/EBPβ exhibited strong DNA binding activity in IL-10-producing GM/DCs but not in IL-12p70 producing IL-4/DCs. These data clearly indicated that C/EBPβ expression and DNA binding activity are involved in regulating immune-regulatory genes of tolerogenic GM/DCs.

C/EBPβ requires the phosphorylation of two residues for its optimal transcriptional activity [40,48,53]. Its phosphorylation of Thr188 is mediated by p38 MAPK or by ERK1/2, depending on cell type [48]. We found that p38 MAPK was involved in phosphorylation of Thr188 in GM/DCs, which also entailed an increased DNA binding activity. The drastic reductions of C/EBPβ phosphorylation and its DNA binding activity in the presence of the p38MAPK inhibitor correlated with a decrease in CREB DNA binding activity, which is known to regulate C/EBPβ expression [54]. The other phosphorylation sites of C/EBPβ are mediated by GSK3 at residues Thr179 and Ser184. These covalent modifications allow C/EBPβ to undergo conformational changes needed to bind DNA [40,48]. GSK3 is known to phosphorylate several other transcription factors such as CREB and AP-1, which regulate IL-10 gene expression [55,56]. In agreement with these reports, we found a robust phosphorylation of CREB at Ser129 and Ser133 in GM/DCs but not in IL-4/DCs, indicating high GSK3 activity in tolerogenic GM/DCs. In immunogenic IL-4/DCs, CREB phosphorylation at Ser129 was less important, suggesting lower GSK3 activity in these cells. The low levels of CREB phosphorylation of Ser129 in IL-4/DCs could be attributed to the fact that IL-4 is an important activator of the PI3K/Akt pathway [57], which is responsible for phosphorylation and inactivation of GSK3 [58]. 

We have previously reported that MEK1/2 inhibition in GM/DCs resulted in inhibition of AP-1 DNA binding activity and a 50% decrease of IL-10 production. These observations suggested that other pathways were involved in regulating IL-10 production in GM/DCs [8]. GSK3 has been reported to play a central role in determining the nature and magnitude of pro- versus anti-inflammatory cytokine production [39,59]. Our data showed that the inhibition of GSK3 activity increased IL-10 secretion whereas IL-12 production was significantly reduced in LPS-stimulated GM/DCs. These results are in agreement with previous studies that reported that GSK3 inhibition triggered increases in IL-10 production but decreases in IL-12 production by human monocytes, DCs and macrophages in response to TLR agonists [39,59,60,61]. Similarly, it has been reported that GSK3 inhibition increased CREB DNA binding activity that resulted in suppression of pro-inflammatory cytokines production but increased IL-10 secretion. These combined effects favored protection of mice from endotoxin shock [39]. Inhibition of GSK3 in monocyte-derived DCs resulted in an increased surface expression of costimulatory molecules [62,63]. In agreement with these reports, our results showed that GSK3 inhibition led to increased levels of expression of CD80 and CD86 in the case of LPS stimulated GM/DCs whereas inhibition of p38 MAPK activity had a minor effect on LPS-stimulated GM/DCs maturation status. These observations could be attributed to low levels of costimulatory molecules expressed on semi-mature GM/DCs. In addition, other groups have shown that RelB was involved in expression of costimulatory molecules in DCs [64,65]. It has been reported that CREB was essential for expression of CD80, CD83, and CD86 in human monocyte-derived DCs [66]. In this respect, we found that GSK3 inhibition in GM/DCs had no effect on CREB DNA binding activity but significantly reduced DNA binding of C/EBPβ. These observations suggested the involvement of GSK3β-C/EBPβ in regulating phenotype and IL-10/IL-12 production by GM/DCs. 

Classical C/EBP binding sites are present in many cytokine promoters such as those of IL-12p40, GM-CSF and IL-6 genes whereas CREB regulates the expression of genes such as IL-10. We found that inhibition of p38 MAPK in GM/DCs led to a drastic reduction in both CREB and C/EBPβ DNA binding activities, as well as a loss of IL-10 production. These findings suggest that inhibition of p38 MAPK was likely to target both C/EBPβ and CREB factors, and the latter is also known to control IL-10 production. Therefore, the presence of residual IL-10 is not unexpected in C/EBPβ^−/−^ GM/DCs, as CREB would remain unaffected (as would other transcription factors). However, p38 MAPK inhibition led to an increased production of IL-12p70 that was comparable to that of IL-4/DCs. Our data are in line with the report that showed that C5aR inhibited the pro-inflammatory potential of a very pro-inflammatory human DC subset (slanDCs) and induced IL-10 production by initiating and prolonging ERK and p38 MAPK phosphorylation as well as CREB phosphorylation [67]. Furthermore, inhibition of ERK1/2 or p38 MAPK phosphorylation or inhibition of signal transduction upstream of CREB1 (via MSK1/2 inhibition) abrogated the ability of C5aR to induce IL-10 gene expression in LPS-stimulated moDCs. Another report has shown that the p38 MAPK-CREB axis mediated IL-10 secretion in TLR2 and PSM-treated DCs and that resulted in enhanced activation of p38 MAPK-CREB-IL-10 axis. Of significance, this response was prevented by inhibition of p38 MAPK, suggesting the involvement of the p38 MAPK-CREB axis in IL-10 production induced by PSMa3 [68].

Our study showed, to the best of our knowledge, for the first time that C/EBPβ was responsible for the low expression of the costimulatory molecules in tolerogenic GM/DCs. This observation was corroborated by increased expression of costimulatory molecules in GM/DCs generated from C/EBPβ^−/−^ mice. We have previously reported that GM/DCs expressed low levels of CD80 and CD86 but high levels of PDL-1. They also produced high amounts of IL-10 while reducing their ability to activate autoreactive T cells. These GM/DCs induced Treg differentiation, suggesting that these cells possess the signature of tolerogenic DCs [7,8]. Here, we found high levels of expression and strong DNA binding activity of C/EBPβ in tolerogenic GM/DCs as compared to immunogenic IL-4/DCs. Our findings were in agreement with the report that showed that tumors induced tolerogenic and immunosuppressive MDSCs, and that the tumor-derived soluble factor GM-CSF controlled this immunosuppression in a C/EBPβ-dependent manner [69]. Since in our bone marrow derived DCs, 90% of non-adherent cells expressed moderate to high levels of CD11c/MHC-Class II and less than 10% were Gr1 positive, we do not exclude the possibility that the deletion of C/EBPβ in myeloid cell subpopulations may also contribute to IL-10/IL-12p70 regulation. In addition, a recent study has shown that the lymphoma-promoting tolerogenic function of IL-10 produced by DCs was regulated by the C/EBPβ transcription factor [50]. This study further showed that, C/EBPβ controlled-lymphoma-associated C/EBPβ competent DCs expressed high levels of IL-10 and IL-6. In contrast, lymphoma-associated C/EBPβ-deficient DCs produced less amounts of these cytokines [50]. To explore the function of C/EBPβ in GM/DCs, we focused on genes that were informative for immunoregulatory as opposed to inflammatory function. We selected IL-10, IL-6, and IL-12 cytokines and costimulatory molecules as targets of our investigations. Interestingly, we found lower levels of expression of IL-10 mRNA and reduced secretion after a 24 h stimulation with LPS in GM/DCs of C/EBPβ^−/−^ mice compared to C/EBPβ^+/+^ mice. These observations suggested that C/EBPβ was also necessary for gene expression and production of IL-10. In the context of autoimmune diabetes, pDCs have been shown to be essential to initiate the development of the disease whereas pDCs depletion induced diabetes protection [70,71]. We have previously have reported that conventional tolerogenic DCs are major contributors to diabetes resistance [72]. Of interest, pDCs were drastically diminished in diabetes-resistant mice and their conventional DCs express low level of IRF8 as compared to DCs of diabetic mice (Zerif E. et al. unpublished data). It is therefore tempting to hypothesize that tolerogenic DCs function in diabetes-resistant mice express high levels of active C/EBPβ as we observed in the case of tolerogenic GM/DCs as compared to IL-4/DCs. C/EBPβ phosphorylation and DNA binding in conventional DCs of diabetes-resistant NOD mice and diabetic NOD mice is currently under investigation in our laboratory.

Data reported here led us to conclude that C/EBPβ was critical for production of IL-12 and IL-6 since their production was drastically reduced in C/EBPβ^−/−^ GM/DCs. In this connection, previous reports have shown that IL-6 production was not affected in activated C/EBP^−/−^ macrophages and that C/EBPβ compensated the lack of C/EBPβ for IL-6 production [30,31]. Since LPS-stimulated GM/DCs produced IL-6 in the absence of C/EBPβ DNA binding activity, this finding suggested that the inhibition of IL-6 production observed in C/EBPβ^−/−^ GM/DCs was not compensated by other C/EBP isoforms. However, our data do not exclude the possibility that C/EBP is not involved in IL-6 production by tolerogenic DCs. Importantly, we found that inhibition of p38 MAPK in GM/DCs or C/EBPβ^−/−^ GM/DCs have greater capacity to induce CD4^+^ T cells proliferation and enhanced IFNγ production. High production of IFNγ by CD4^+^ T cells could be the results of marked reduction of IL-10 and IL-6 produced by C/EBPβ^−/−^ GM/DCs. Indeed, IL-10 and IL-6 have been reported to be limiting factors for Th1 development response even in the presence of IL-12 [73,74,75]. Our findings are also supported by a study that used adoptively transferred OT-II CD4^+^ T cells into tumor-bearing mice that showed that lymphoma-educated C/EBPβ competent DCs reduced OT-II CD4^+^ T cell proliferation and that inhibition of OT-II T cell proliferation was reverted in the presence of C/EBPβ^−/−^ DCs [50]. Furthermore, it has been reported that C/EBPβ plays an essential role in limiting immunosuppressive function such as inhibiting T cell proliferation and IFNγ production [49]. These authors found that C/EBPβ^−/−^ MDSC cells produce less IL-10 and induce a strong CD4^+^ T cell proliferation and IFNγ production, whereas C/EBPβ overexpression repress CD4^+^ T cell proliferation and activation, as well as their IFNγ production. Data reported here clearly highlight the fact that C/EBPβ is a key transcription factor that governs tolerogenic phenotype and function of GM/DCs, which is critical in tolerance induction within the context of autoimmunity.

## 5. Conclusions

In conclusion, our study has shown that C/EBPβ is a master regulator of tolerogenic DCs function and has identified a critical role for the p38 MAPK-C/EBPβ pathway in the regulation of IL-10 and IL-12p70 production, as well as the maturation status of bone marrow-derived tolerogenic semi-mature GM/DCs. In particular, we have identified key signaling molecules involved in the regulation of important features of tolerogenic DCs, namely p38 MAPK and GSK3. Clinical implications may emerge from these observations such as targeting pathways that lead to C/EBPβ activation in DCs. This experimental approach could help to improve tolerogenic DCs function and may be used as a basis to design efficient therapy to prevent autoimmune diseases such as type I diabetes.

## Supplementary Materials

The following are available online at http://www.mdpi.com/2073-4409/7/12/256/s1, Figure S1: Cellular localization of C/EBPβ in GM/DCs and IL-4/DCs, Figure S2: Effect of GSK3 inhibitor (LiCl) on IL-10 and IL-12p70 production by GM/DCs, Table S1: Sequences of the primers used for real time PCR analysis.
