# Supplementary material for: Role of the p38 MAPK/C/EBPβ Pathway in the Regulation of Phenotype and IL-10 and IL-12 Production by Tolerogenic Bone Marrow-Derived Dendritic Cells
Source: Cells. 2018 Dec 7;7(12):256. doi: 10.3390/cells7120256 (PMC6316502; doi:10.3390/cells7120256)
Supplement: Supplementary file 1 [file cells-07-00256-s001.pdf]

Genes of interest	Forward primers	Reverse primers	Tm (oC)
IL-12p35	CTCCTAAACCACCTCAGTTTGGCCAGGGTC	TAGATGCTACAAGGCACAGGGTCA TCATC	60
IL-12p40	CACTCATGGCCATGTGGGAGCTGGAGAAAG	TCCGGAGTAATTTGGTGCCTTCACACC TCAG	60
IL-10	TACCTGGTAGAAGTGATGCC	CATCATGTATGCTTCTATGC	64
β-Actin	ACCCACACTGTGCCCATCTA	TCATGGATGCCACAGGATTC	55-65

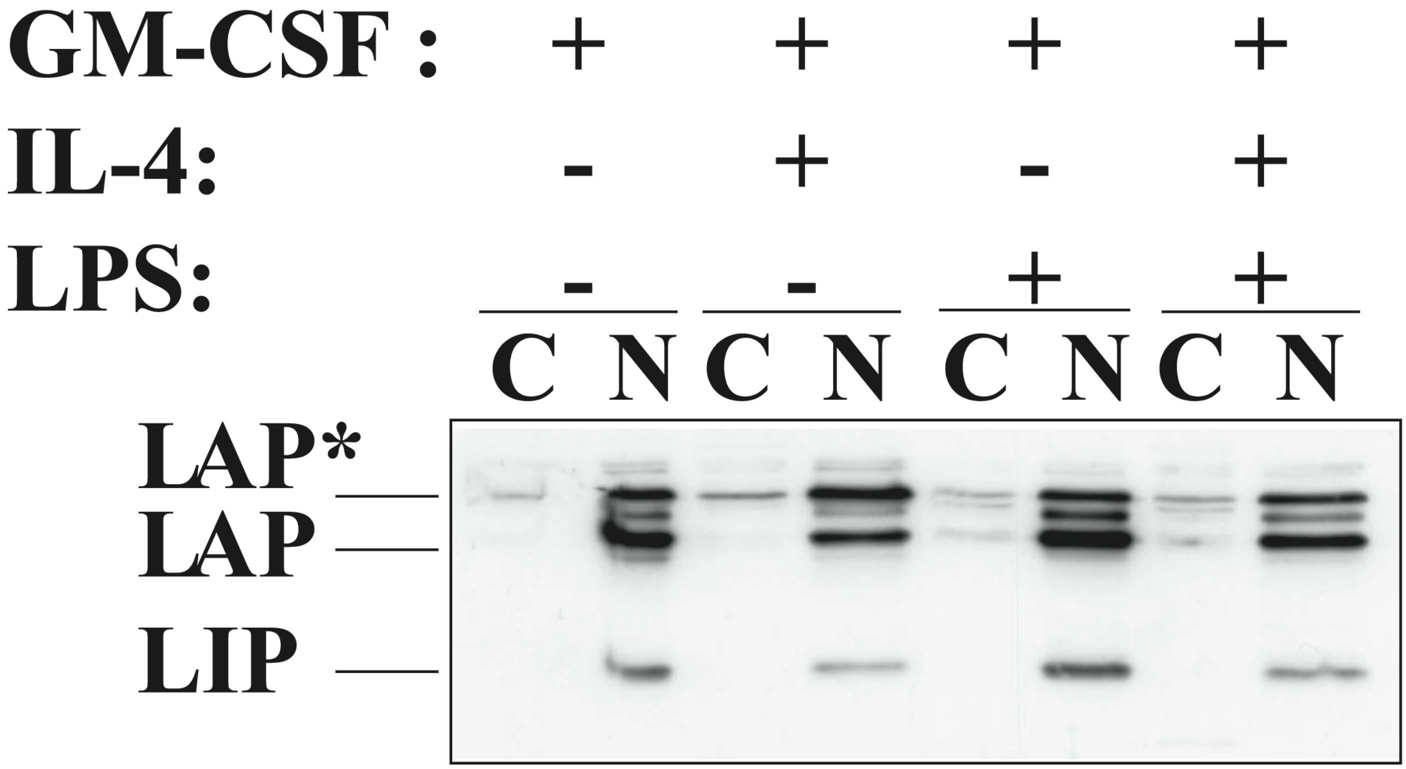

Guindi et al. Suppl. Figure 1

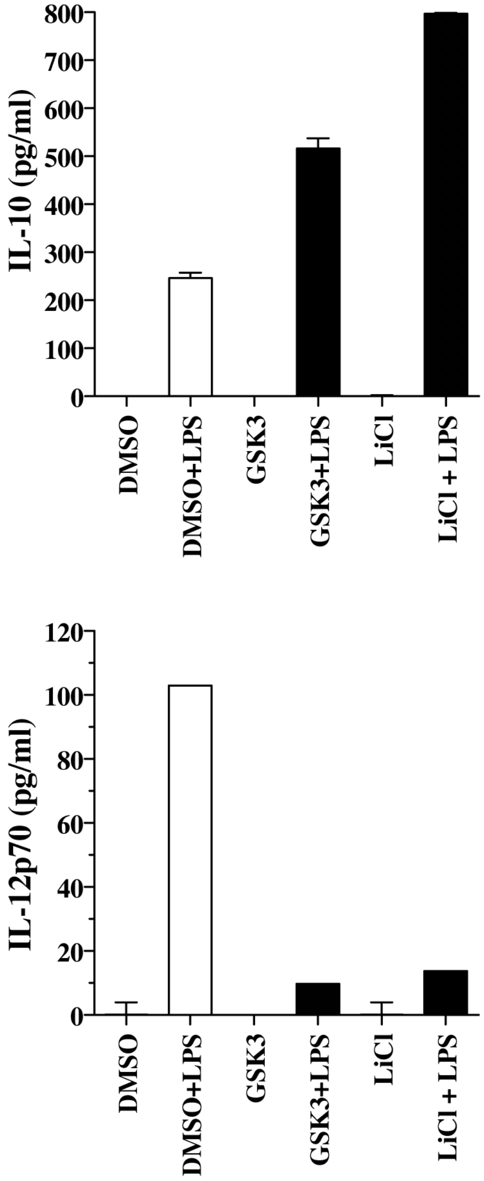

Guindi et al. Suppl. Figure 2
